# Upper body posture in Latin American dancers: a quantitative cross-sectional study comparing different postures

**DOI:** 10.1186/s13102-023-00672-w

**Published:** 2023-04-25

**Authors:** Eileen M. Wanke, Manja Mörl-Kreitschmann, Fabian Holzgreve, David Groneberg, Daniela Ohlendorf

**Affiliations:** 1 Institute of Environmental, Social and Occupational Medicine, Goethe University, Theodor-Stern-Kai 7, Haus 9a, 60590 Frankfurt am Main, Germany; 2 Institute of Sports Science, Goethe University, Frankfurt Am Main, Germany

**Keywords:** Dance sport, Torso position, Three-dimensional back scan, Dance-specific positions

## Abstract

**Background:**

In Latin American dance sport (LD), the shoulder girdle and the torso area are particularly stressed due to the dance style specific requirements. The aim of the study was to define differences in various dance specific upper body postures in Latin American dancers and to show gender-specific differences.

**Methods:**

Three dimensional back scans were performed in n = 49 dancers (28 f/21 m). Five typical trunk positions in Latin American dance (habitual standing and 4 dance specific positions, P1–P5) were compared with each other. Statistical differences were calculated using the Man-Whitney U test, Friedmann test, Conover-Iman test and a Bonferroni-Holm correction.

**Results:**

Significant gender differences were found in P2, P3 and P4 (*p* ≤ 0.01–0.001). In P5, the frontal trunk decline, the axis deviation, the standard deviation of the rotation, the kyphosis angle and the shoulder as well as the pelvic rotation were also significantly different. The comparison of the postures showed significant differences between postures 1–5 (*p* ≤ 0.01–0.001) in the males*,* (scapular height, right and left scapular angles and pelvic torsion). Similar results were observed for the female dancers, with only the parameters of frontal trunk decline with the lordosis angle as well as the right and left scapular angles being non-significant.

**Conclusions:**

This study is an approach to better understand the involved muscular structures in LD. Performing LD changes the static parameters of the upper body statics. Further projects are needed to analyse the field of dance even more thoroughly.

## Introduction

Latin American dance sport (LD) is a technical-compositional sport that has been constantly developed and has become a recognised competitive sport with high demands in terms of coordination, conditional skills and agility [1, 2]. In competitions, the Rumba, Cha-Cha-Cha, Samba, Paso Doble and Jive are performed by a dance couple [3]. Due to the technical requirements, not only the shoulder girdle area and the lower extremities are stressed, but also the torso area because of the specific body movements in this dance style [4]. Although several studies have investigated the upper body posture in different sport-specific tasks [5, 6], there have only been few studies on the upper body posture in relation to compositional sports in general, and in the Latin American dance style up to now [7, 8]. Mulhearn and George [9] found that up to 80% of gymnasts exhibited a sway-back posture and concluded that this is a common phenomenon in gymnastics. Mahdavie et al. [10] studied the thickness of the multifidi lumboris muscles in relation to sway-back posture in gymnasts and found significantly lower muscle thicknesses f in the sway-back group compared to the control group. Liiv et al. [11] studied 60 people to determine the somatotype of standard dance and Latin American dance. Standard dancers stood out mainly because of their longer torsos, while Latin American dancers were more mesomorphic. Kruusamäe et al. [12] measured 30 couples and compared them with track and field athletes with a significantly smaller S-curve of the spine found in the dancers. Both, male and female dancers showed a lower lordosis angle than the track and field athletes, while the female dancers also had a lower kyphosis angle. Wanke et al. [13] compared high-class formation dancers of two performance classes with a control group. They described unilateral higher isometric strength values as well as shortened muscle groups, both caused by the effects of formation dancing. Up to now, there have been little or no studies allowing a comparison with the normal values for upper body postures described by Ohlendorf et al. [14, 15, 18, 19].

In Latin American dance, there are requested upper body postures that correspond to the technique model. A distinction can be made between European and African standards of posture in dance [16]. The basic requirements for the European modern standard dances are an erect spine and upright stance and gait; neither the shoulders nor the thorax are strongly raised with the natural S-shape of the spine being maintained [2], while the sternum and rib arches are raised [16]. The body's centre of gravity is vertically above the centre of the standing surface [2] with the entire posture slightly tilted forwards [2]. This European posture applies to the Cha-Cha-Cha and Rumba dances and serves as the basis for the dance posture used in this study. The musculature of the dancer is indispensable for strength endurance, but also provides the body with good mobility and extreme positions [4, 17]. The abdominal and back muscles are responsible for all trunk movements, especially the so-called internal body movements required for fine tuning and good muscular coordination. A high degree of flexibility is necessary to be able to perform all required internal body movements and poses (e.g. sway, shape or twists) [4].

The aim of the study was to examine whether Latin American dance sport results in changes in the area of upper body statics compared to the normal population. At that, the study aimed to define differences in the upper body statics of various postures in Latin American dancers and also to show gender-specific differences. The structure of the study was based on Ohlendorf et al. [18, 19].

## Methods

Before this quantitative cross-sectional study began, a positive ethical vote was obtained from the ethics committee of the Goethe University (No. 519/15). Within the framework of the study, a total of n = 49 (21 m, 28 f) active Latin American dancers were measured using video raster stereography. In order to ensure comparability of the results (e.g. age, technical quality of the dancers), inclusion criteria were defined:

- voluntary participation;
- aged between 18 and 40 years;
- at least 2 years of tournament experience;
- a training workload of at least four hours per week.

Exclusion criteria included:

- an acute injury or a diagnosed sports injury in the spinal area within the last 6 weeks, provided this resulted in a break from sport of more than 2 weeks;
- pregnancy.

**Fig. 1:**
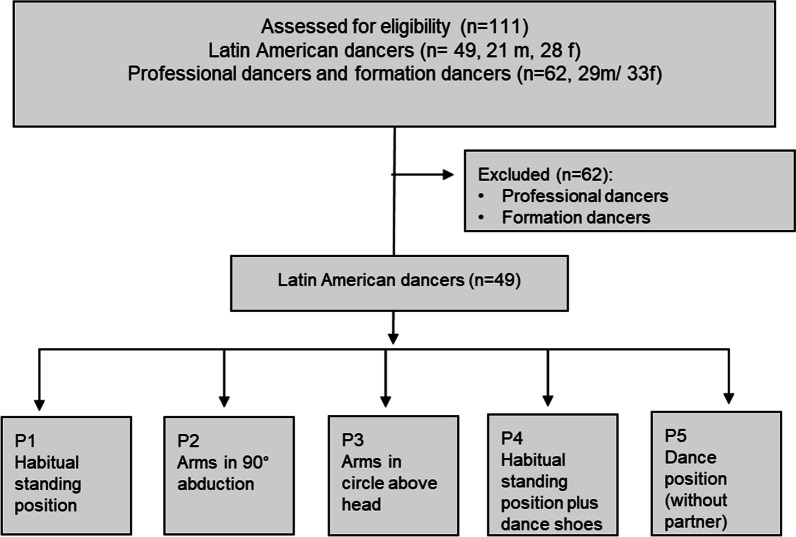
Investigation procedure chart

A back scanner (model *MiniRot Kombi System* from *ABW GmbH Frickenhausen, Germany*) was used as the measurement system. This projects a moving strip grid (zebra pattern) onto the back of the test person using video raster stereography, thus recording an image of the back and calculating various angles (measuring frequency: 50 Hz). The resulting scan represents the surface of the back, three-dimensionally, on the basis of a two-second image capture which includess the calculation of the coordinates of the marker points. A system error of < 1 mm is specified by the manufacturer (manufacturer's information). This scanner examination is a reliable measurement method that is also gentle on the body [18-22]. A detailed description of all evaluation parameters can be found in Ohlendorf et al. [19]. The full investigation procedure is shown in Fig. 1.

The contact to the participants was established through the national and regional dance coaches. Participation in this study was on a voluntary basis. The study was conducted at the training site before, during or after a training session. Prior to the start of the study, the participants were informed in detail on the procedure. Informed consent was obtained from all participants involved in the stud before the start. A total of five measurement conditions were tested, of which postures 1, 2 and 3 were recorded barefooted with postures 4 and 5 in dance shoes:

- Habitual posture 1 (P1): in this neutral-zero method, the subjects stood with their arms hanging down in what they felt was a normal posture.
- Posture 2 (P2): the arms were extended laterally in 90° abduction.
- Posture 3 (P3): a circle with both arms was formed above the head.
- Posture 4 (P4): habitual posture in dance shoes analogous to posture 1.
- Posture 5 (P5): the subjects extended their lead arm in 90° anteversion and the free arm in 90° abduction. The subjects were asked to assume their dancing posture, including the corresponding back and spine alignment. The decision to rest the body weight either on the left or right foot was according to the Latin dance technique guidelines (Rumba technique). As to the technical convention, a Rumba dance sequence begins with the upbeat step and a basic step. The dancer stands on the right foot with the left leg extended forwards. In Latin dance, the leading arm is not held at shoulder height but at hip height; the exact height is derived from the size difference between the dancing partners. For the sake of standardisation, this deviation from dance practice was tolerated.

**Fig. 2:**
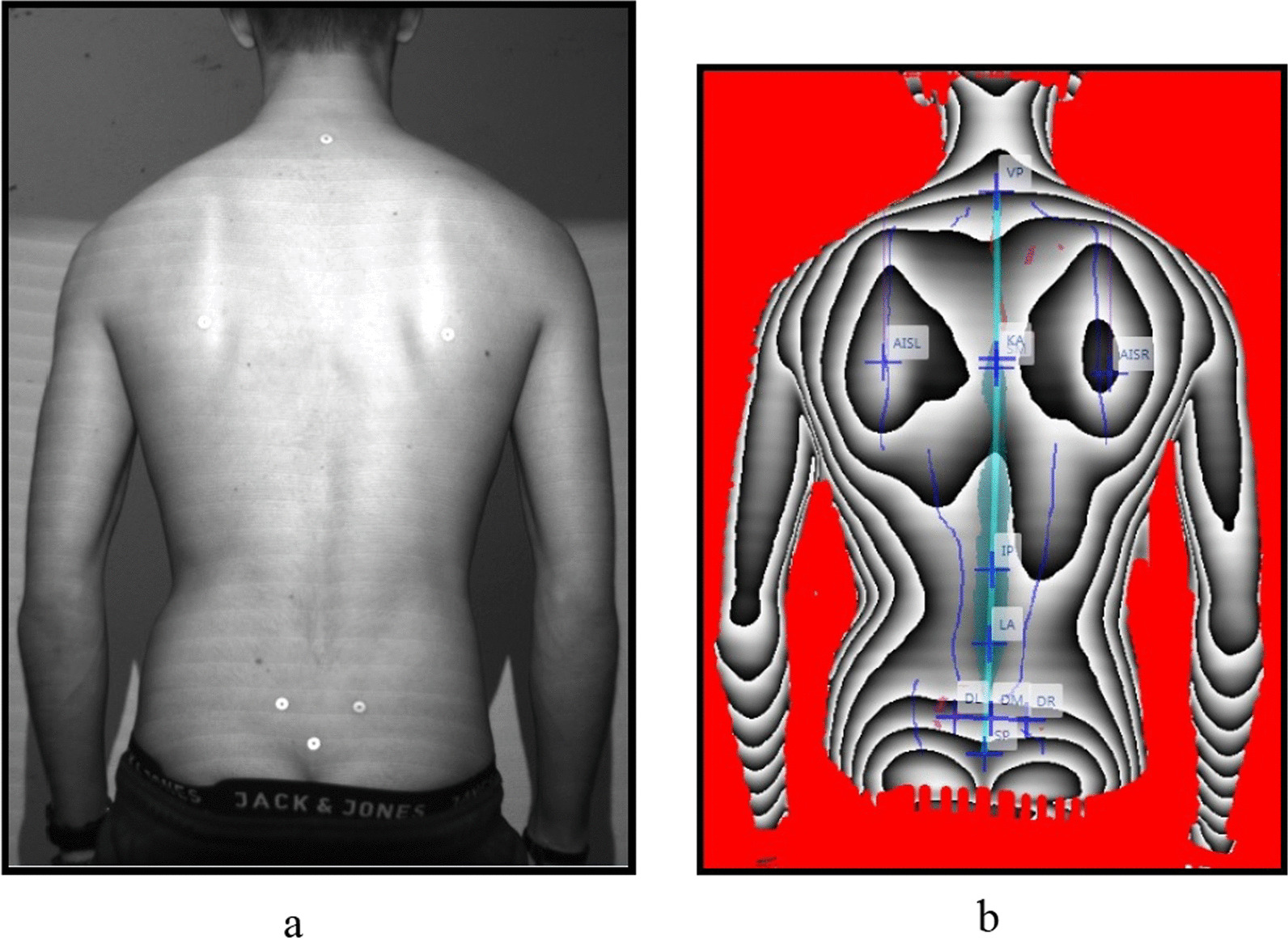
**a** Marker position of a male dancer in P1. **b** Phase image of a male dance in P1

**Fig. 3:**
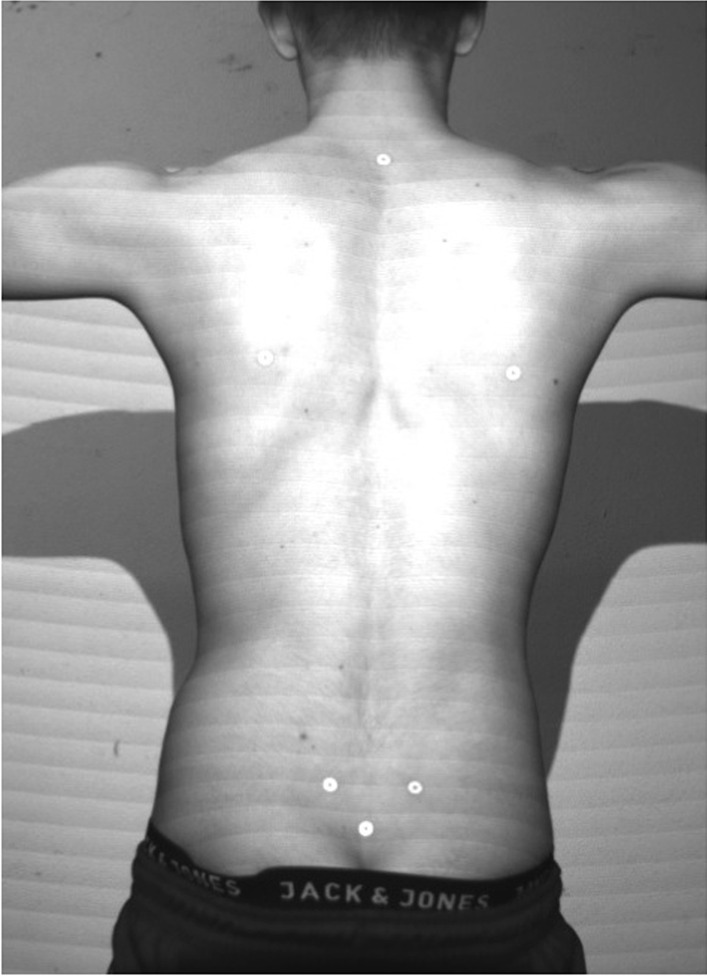
Marker positions in P2 of a male dancer

**Fig. 4:**
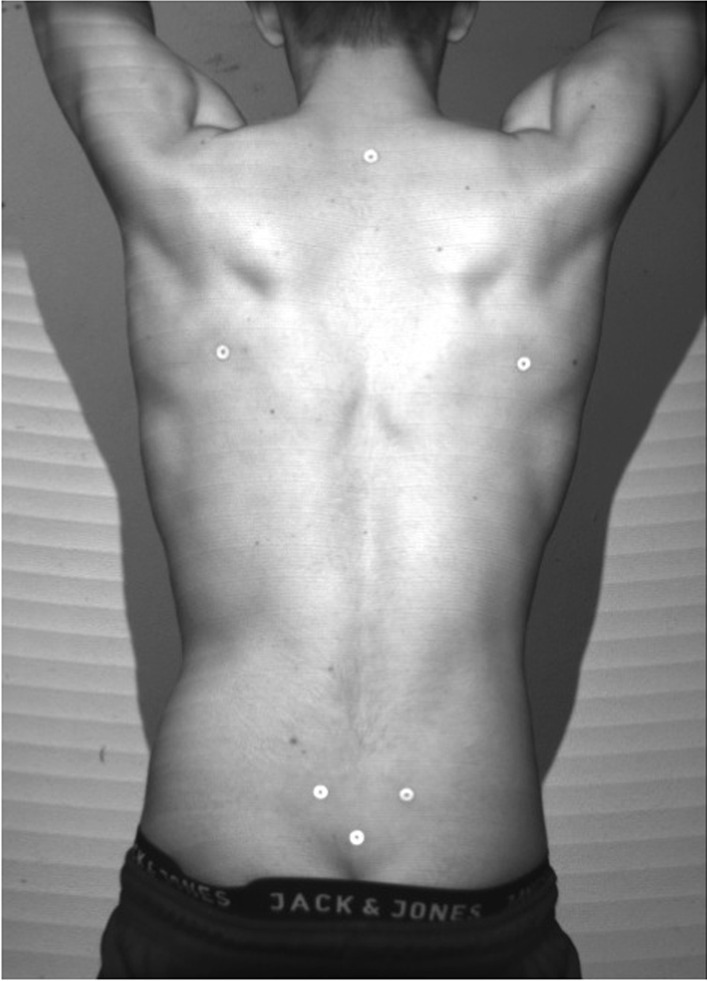
Marker positions in P3 of a male dancer

**Fig. 5:**
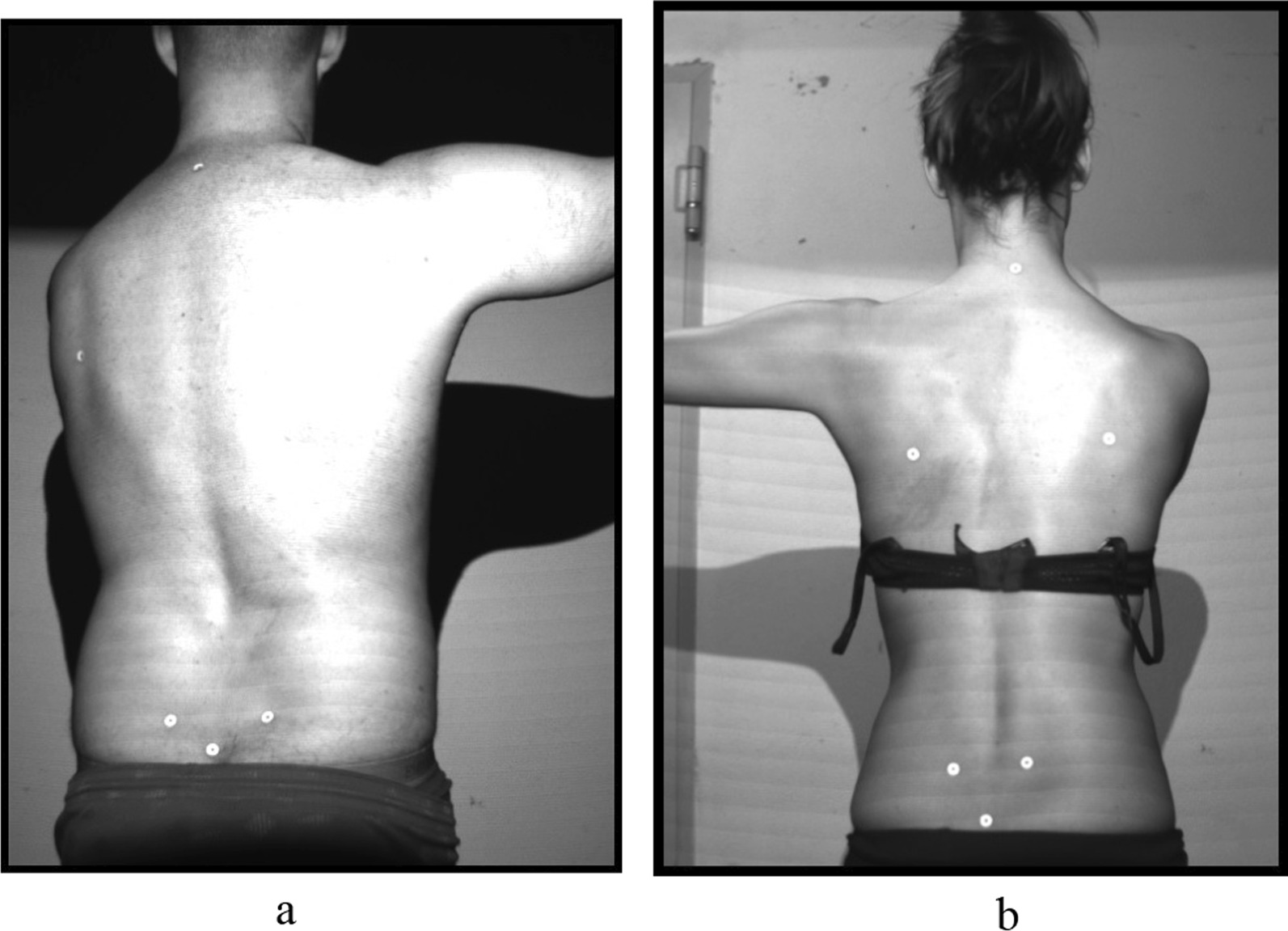
**a** Marker position in P5 of a male dancer. **b** Marker positions in P5 of a female dancer

In order to be measured, the subjects stood with their bare backs about 90 to 100 cm in front of the scanner. A maximum of nine recording attempts were carried out per subject and posture. The test persons were assigned anatomical marker points on the *vertebrae prominens* (*C7*), the lower *angulus scapulae on the* right and left (AIS R/AIS L), the *spinae iliacae posteriores superiores SIPS* and the *sacrum point* which were used for calibration by the scanner. The recordings were made of the individuals without their dance partners. Due to time optimisation, no randomisation was carried out. The measurements were taken in the following order:

1. P1: habitual posture without shoes (Fig. 2a, b);
2. P2: ballet posture arms sideways in 90° abduction without shoes (Fig. 3);
3. P3: ballet posture arms above head without shoes (Fig. 4);
4. P4: habitual posture in dance shoes;
5. P5: standardised dance posture in dance shoes (Fig. 5a, b).

The 20 calculated parameters were the torso length D (distance between C7 and SIPS), torso length S (distance between C7 and *sacrum point*), frontal torso slope, axial deviation, sagittal torso slope, thoracic bending angle, lumbar bending angle, kyphosis angle, lordosis angle, standard deviation of lateral deviation, standard deviation of rotation, scapula distance, scapula stance, scapula rotation, shoulder stance angle left/right, pelvic distance, pelvic stance, pelvic torsion and pelvic rotation.

### Statistical evaluation

All data were tested for normality using the Kolmogorv-Smirnov test. As the data sets were not normally distributed, non-parametric tests were used. The gender comparison was carried out with the Man-Whitney U test, while the posture comparison was achieved with the Friedmann test, including multiple testing by the Conover-Iman test. All p-values were subjected to a Bonferroni-Holm correction. From a descriptive point of view, a range of − 1° + 1° was considered neutral.

## Results

The anthropometric data of the male and female dancers are shown in Table 1. None of the male test persons were underweight, while *n* = *16* dancers were of normal weight and *n* = *5* were even overweight. Among the female dancers, *n* = *4* were underweight, *n* = *22* were of normal weight and *n* = *2* had a weight slightly above normal weight.

**Table 1:** Anthropometric data of the dancers

	Age (in years)	BMI kg/m^2^	Right-handed	Left-handed	Training experience (in years)	Training frequency (in hours)	Heel height of the shoe (in cm)
Male	28.0 ± 5.0	23.1 ± 3.2	17	4	11.0 ± 6.0	9.0 ± 4.0	3.55 ± 0.26
Female	28.0 ± 5.0	20.8 ± 2.1	24	4	11.0 ± 5.0	9.0 ± 4.0	6.97 ± 0.79

### Posture comparison

A posture comparison was carried out in male and female dancers shown in Table 2. All males*,* apart from the axis decline, the scapular height, right and left scapular angles and pelvic torsion, showed significant differences between postures 1–5, according to the Friedman test (*p* ≤ 0.01 or 0.001). The subsequent multiple pair comparisons revealed various significances between the two postures (*p* ≤ 0.05–0.001), whereby these were present between the three different barefoot positions, the two positions with dance shoes and also between the barefoot and shoe positions in all three areas (spine, shoulder and pelvis). With regard to the descriptive data of the significant pair comparisons, it should be noted that they always changed according to the posture adopted.

**Table 2:** Comparison of the postures

		Position 1		Position 2		Position 3		Position 4		Position 5	
		Male	Female	Male	Female	Male	Female	Male	Female	Male	Female
*Spine parameter*											
Trunk length D (mm)	Median	492.58^c^	439.17^c^	490.90^c^	436.76^c^	485.01^c^	427.16^c^	491.92^c^	439.96^c^	475.34^c^	429.70^c^
	1st quartile	472.33	431.78	475.78	431.19	466.06	422.51	473.32	434.41	458.14	421.12
	3rd quartile	500.24	449.82	499.74	445.35	490.82	439.11	496.23	450.49	486.02	441.99
Trunk length S (mm)	Median	523.41^c^	472.90^c^	524.32^c^	468.53^c^	516.20^c^	459.78^c^	524.44^c^	473.00^c^	507.76^c^	463.91^c^
	1st quartile	507.89	465.32	510.25	462.24	503.85	455.05	507.98	465.81	484.17	454.89
	3rd quartile	538.81	483.64	538.46	484.64	529.05	473.33	537.60	487.51	522.86	477.60
Sagittal trunkdecline (°)	Median	− 2.48	− 2.08	− 2.59	− 2.36	− 0.69	0.41	− 2.56	− 2.06	− 2.69	− 0.97
	1st quartile	− 3.53	− 2.78	− 3.46	− 3.10	− 1.23	− 0.87	− 3.81	− 3.47	− 4.52	− 3.74
	3rd quartile	− 1.34	− 0.99	− 1.14	− 0.93	1.05	1.42	− 1.62	− 0.71	− 1.53	1.28
Frontal trunk decline (°)	Median	− 0.41	− 0.58	− 0.26	− 0.43	− 0.26	− 0.60	− 0.30	− 0.28	− 2.27^c^	2.97^c^
	1st quartile	− 0.69	− 0.89	− 0.91	− 0.98	− 0.91	− 0.95	− 0.82	− 0.90	− 3.36	0.17
	3rd quartile	0.54	0.24	0.54	0.16	0.39	0.30	0.63	0.17	− 1.09	3.81
Axis decline (°)	Median	− 1.06	− 0.61	− 0.77	− 0.54	− 1.48	− 0.42	− 1.21	− 0.54	− 0.61^c^	4.98^c^
	1st quartile	− 1.68	− 1.49	− 1.98	− 1.30	− 2.16	− 1.36	− 2.17	− 1.61	− 2.22	2.77
	3rd quartile	− 0.24	0.19	− 0.13	0.39	− 0.28	0.74	− 0.41	0.47	2.47	7.06
Thoracic bending angle (°)	Median	12.64^a^	9.92^a^	12.26	10.30	10.47	9.43	12.99	10.89	10.98	9.91
	1st quartile	9.92	8.22	11.47	6.99	9.74	5.51	10.21	8.35	8.95	6.59
	3rd quartile	14.77	12.65	16.65	14.14	11.77	14.85	13.89	13.41	12.86	12.40
Lumbar bending angle (°)	Median	8.25^c^	12.03^c^	6.60^b^	10.72^b^	7.73^b^	10.48^b^	8.25^b^	10.70^b^	7.06^c^	11.81^c^
	1st quartile	6.79	9.82	5.72	8.31	6.71	8.58	6.14	9.47	5.28	9.37
	3rd quartile	11.33	14.38	10.45	12.51	10.99	12.93	10.63	14.33	9.28	13.75
Standard deviation lateral deviation (°)	Median	3.58	3.58	4.46	4.48	3.61	4.21	4.26	4.10	8.61	7.80
	1st quartile	2.69	2.04	3.70	2.97	2.84	2.85	2.64	2.64	6.77	5.22
	3rd quartile	5.33	5.39	5.34	5.54	5.50	5.43	5.58	6.58	10.00	9.74
Standard deviation rotation (°)	Median	3.83	3.57	3.25	3.36	3.22	3.16	3.77	3.62	21.49^c^	10.26^c^
	1st quartile	2.08	2.47	2.73	2.42	2.12	2.66	2.26	2.45	17.26	8.30
	3rd quartile	4.66	4.95	5.66	4.53	4.17	3.82	5.77	4.43	27.76	12.70
Kyphosis angle (°)	Median	44.30	42.08	39.13	44.08	35.53	33.82	40.73	40.72	34.69^a^	43.07^a^
	1st quartile	36.82	33.26	32.20	34.19	29.93	27.71	36.23	32.10	31.80	33.06
	3rd quartile	47.81	55.69	44.84	49.13	43.01	40.64	48.63	47.72	40.90	47.07
Lordosis angle (°)	Median	30.87^c^	42.47^c^	28.61^c^	44.79^c^	32.19^b^	41.23^b^	31.26^c^	40.35^c^	31.34^c^	44.31^c^
	1st quartile	23.69	38.28	25.68	38.23	26.78	31.90	24.65	35.32	27.17	38.86
	3rd quartile	37.15	59.98	37.97	56.71	40.31	52.99	34.87	52.56	39.89	52.17
*Shoulder parameter*											
Scapular distance (mm)	Median	190.26^c^	161.71^c^	179.51^c^	157.88^c^	210.34^c^	179.11^c^	190.30^c^	165.77^c^	196.55^c^	168.31^c^
	1st quartile	178.43	152.44	172.03	146.75	198.23	169.75	175.53	152.61	177.38	162.76
	3rd quartile	200.10	173.71	202.03	167.87	224.74	189.75	196.12	173.74	215.90	176.99
Scapular height (mm)	Median	1.59	0.33	3.81	1.01	2.84	1.03	2.78	0.03	6.88	3.26
	1st quartile	− 1.92	− 3.17	− 3.42	− 2.44	− 0.36	− 2.50	− 0.82	− 2.37	0.65	1.14
	3rd quartile	8.26	5.72	7.82	5.36	6.17	4.29	7.19	5.59	9.90	7.47
Scapular rotation (°)	Median	2.35	2.41	2.72	3.35	0.84	2.40	3.02	2.01	19.60^c^	− 4.12^c^−
	1st quartile	0.81	0.61	− 1.10	− 0.89	− 0.33	0.61	0.92	0.68	13.24	10.06
	3rd quartile	4.98	4.66	4.02	4.86	2.57	3.80	5.54	4.00	23.68	− 1.00
Right scapular angle (°)	Median	30.93	27.99	22.42	21.87	12.59	23.96	31.31	27.36	26.90	31.17
	1st quartile	28.42	24.79	20.33	15.16	6.85	13.07	29.62	21.63	20.25	23.90
	3rd quartile	32.05	32.48	27.40	27.07	15.52	39.88	32.79	34.97	32.59	34.81
Left scapular angle (°)	Median	30.27^b^	28.97^b^	22.91	20.72	22.56	24.39	29.44	29.36	32.23^c^	23.82^c^
	1st quartile	28.42	22.33	18.73	17.27	12.34	19.92	26.94	25.09	24.82	16.99
	3rd quartile	35.70	30.29	25.26	26.74	43.61	29.39	35.27	33.86	41.43	27.50
*Pelvis parameter*											
Pelvic distance (mm)	Median	87.98	82.27	87.05	82.06	88.37	81.96	87.29	81.58	86.71	81.50
	1st quartile	79.58	75.78	77.93	75.68	78.20	75.55	78.30	75.43	79.56	76.18
	3rd quartile	93.85	88.56	94.47	88.30	94.94	88.21	95.25	88.94	97.95	88.69
Pelvic height (°)	Median	− 0.75	− 0.21	− 0.54	− 0.17	− 0.75	− 0.20	− 0.67	− 0.10	3.81	3.31
	1st quartile	− 1.86	− 0.84	− 1.81	− 0.98	− 2.15	− 0.88	− 1.94	− 1.08	0.17	0.89
	3rd quartile	0.22	0.76	0.24	0.77	0.23	1.28	− 0.03	0.55	6.17	6.50
Pelvic torsion (°)	Median	0.91	0.43	0.08	− 0.21	1.20	0.12	1.58	0.00	3.17	4.01
	1st quartile	− 1.01	− 2.36	− 0.65	− 2.50	− 0.23	− 2.05	− 1.35	− 2.92	− 0.78	1.61
	3rd quartile	2.78	2.61	3.18	2.33	2.38	2.25	3.52	2.02	8.54	6.52
Pelvic rotation (°)	Median	0.25	0.97	0.32	0.54	0.10	0.46	1.29−	0.77	16.59^c^	4.94^c^
	1st quartile	− 1.48	− 2.25	− 1.47	− 1.52	− 2.76	0.99	0.92	− 2.06	12.52	0.72
	3rd quartile	3.03	2.38	2.59	2.33	2.32	2.31	4.16	2.35	20.69	13.92

A presentation of the *p*-values of the Friedman test per sex and the *p*-values of the Conover-Iman test (including Bonferroni-Holm correction), as well as the *p*-values belonging to both postures

Significant differences between the men and women per posture are marked as follows: a (*p* = 0.05), ^b^(*p* = 0.01), ^c^(*p* = 0.001)

Similar results were observed in the female dancers, with only the parameters of frontal trunk decline, lordosis angle as well as the right and left scapular angles being non-significant (*p* ≥ 0.05).

### Gender comparison

The gender comparison in Table 3 shows significant differences (*p* ≤ 0.05–0.001) in the habitual posture (P1) with regard to the torso length D and S, the thoracic bending angle, lumbar bending angle, lordosis angle, scapula distance and left scapular angle. According to the results, the male dancers were, on average, about 50 mm taller, had median thoracic and lumbar bending angles that were about 2.5° greater and a lordosis angle that was about 11° smaller than in the females. Similarly, the males had an approximately 30 mm wider scapulae distance, with the left shoulder stance angle being marginally more caudal. With regard to the descriptive observation of the values, it can be stated that both male and female dancers had fundamentally symmetrical, balanced upper body statics for the spine, shoulder and pelvic areas. The deviations from the 0° axis were only marginal in the median values.

**Table 3:** Representation of the medians and the 1st and 3rd quartiles of all upper body posture parameters divided into the spine, shoulder and pelvis areas

	*p*-value Friedman test		*p*-value Conover-Iman test (Bonferroni-Holm correction)		Position comparision	
	Male	Female	Male	Female	Male	Female
*Spine parameter*						
Trunk length D (mm)	0.001	0.001		0.001		1 versus 2
			0.001	0.001	1 versus 3	1 versus 3
			0.001	0.001	1 versus 5	1 versus 5
			0.001	0.001	2 versus 3	2 versus 3
				0.001		2 versus 4
			0.01	0.01	2 versus 5	2 versus 5
			0.001	0.001	3 versus 4	3 versus 4
			0.01	0.01		3 versus 5
				0.001	4 versus 5	4 versus 5
Trunk length S (mm)	0.001	0.001		0.05		1 versus 2
			0.001	0.001	1 versus 3	1 versus 3
				0.05		1 versus 4
			0.001	0.001	1 versus 5	1 versus 5
			0.001	0.001	2 versus 3	2 versus 3
				0.001		2 versus 4
			0.001	0.02	2 versus 5	2 versus 5
			0.001	0.001	3 versus 4	3 versus 4
				0.02		3 versus 5
			0.001	0.001	4 versus 5	4 versus 5
Sagittal trunk decline (°)	0.001	0.001	0.001	0.001	1 versus 3	1 versus 3
			0.001	0.001	1 versus 5	1 versus 5
			0.001	0.001	2 versus 3	2 versus 3
						2 versus 4
			0.001	0.001	2 versus 5	2 versus 5
			0.001	0.001	3 versus 4	3 versus 4
			0.001	0.001		3 versus 5
			0.001	0.001	4 versus 5	4 versus 5
Frontal trunk decline (°)	0.001	0.81	0.001		1 versus 5	
			0.001		2 versus 5	
			0.001		3 versus 5	
			0.001		4 versus 5	
Axis decline (°)	0.54	0.001		0.001		1 versus 5
				0.001		2 versus 5
				0.001		3 versus 5
						4 versus 5
Thoracic bending angle (°)	0.001	0.01	0.02		1 versus 3	
			0.04		1 versus 5	1 versus 5
			0.05	0.01	3 versus 4	3 versus 4
Lumbar bending angle (°)	0.001	0.01		0.01		1 versus 2
			0.01		2 versus 3	
				0.05	2 versus 5	
			0.02		3 versus 5	
Standard deviation lateral deviation (°)	0.001	0.001	0.001	0.001	1 versus 5	1 versus 5
			0.01	0.001	2 versus 5	2 versus 5
			0.001	0.001	3 versus 5	3 versus 5
			0.001	0.001	4 versus 5	4 versus 5
Standard deviation rotation (°)	0.001	0.001	0.001	0.001	1 versus 5	1 versus 5
			0.001	0.001	2 versus 5	2 versus 5
			0.001	0.001	3 versus 5	3 versus 5
			0.001	0.001	4 versus 5	4 versus 5
Kyphosis angle (°)	0.001	0.001	0.001		1 versus 2	
			0.001	0.001	1 versus 3	1 versus 3
			0.001		1 versus 5	
			0.001	0.03	2 versus 3	2 versus 3
			0.001		2 versus 4	
			0.03		2 versus 5	
			0.001		3 versus 4	3 versus 5
			0.001	0.02	4 versus 5	3 versus 5
Lordosis angle (°)	0.01	0.16	0.05		2 versus 5	
*Shoulder parameter*						
Scapular distance (mm)	0.001	0.001	0.001	0.001	1 versus 2	
			0.001	0.001	1 versus 3	
			0.02			
			0.02	0.001	1 versus 5	
			0.001	0.001	2 versus 3	
			0.001	0.001	2 versus 4	
			0.001	0.001	2 versus 5	
			0.001	0.001	3 versus 4	
			0.001	0.001	3 versus 5	
			0.001	0.001	4 versus 5	
Scapular height (mm)	0.35	0.01		0.04		1 versus 5
				0.02		3 versus 5
				0.01		4 versus 5
Scapular rotation (°)	0.001	0.001	0.001	0.001	1 versus 5	1 versus 5
			0.001	0.001	2 versus 5	2 versus 5
			0.001	0.001	3 versus 5	3 versus 5
			0.001	0.001	4 versus 5	4 versus 5
Right scapular angle (°)	0.13	0.09				
Left scapular angle (°)	0.46	0.13				
*Pelvis parameter*						
Pelvic distance (mm)	0.05	0.03		0.02	1 versus 4	
			0.04		2 versus 5	
Pelvic height (°)	0.001	0.001	0.02	0.001	1 versus 5	1 versus 5
				0.001		2 versus 5
			0.001	0.001	3 versus 5	3 versus 5
			0.01	0.001	4 versus 5	4 versus 5
Pelvic torsion (°)	0.25	0.001		0.001		1 versus 5
				0.001		2 versus 5
				0.001		3 versus 5
				0.001		4 versus 5
Pelvic rotation (°)	0.001	0.03	0.001		1 versus 5	
			0.03		2 versus 4	
			0.001	0.05	2 versus 5	2 versus 5
			0.01		3 versus 4	
			0.001	0.04	3 versus 5	3 versus 5
			0.01		4 versus 5	

The gender comparison (Table 2) for P2, P3 and P4 showed significant differences (*p* ≤ 0.05–0.001) in the torso length (analogous to posture 1), the lumbar bending angle and the lordosis angle, which were always approximately 3° greater in the median for the lumbar bending angle and approximately 17° greater for the lordosis angle in the females. Furthermore, analogous to posture 1, the male dancers' shoulder blade distance was greater than that of the female dancers in every posture (P2, P3 and P4).

In addition to the significances between the male and female dancers in the other postures (P2, P3 and P4), the frontal trunk decline, the axis deviation, the standard deviation of the rotation, the kyphosis angle, shoulder rotation and pelvic rotation were also significantly different in P5. While female dancers tilted their torso about 3° to the right, the male dancers tilted theirs about 3° to the left (frontal trunk decline). In male dancers, the transverse processes of the vertebrae were found to be rotated about 10° further to the right. In addition to the significantly larger lordosis angle, the kyphosis angle was also significantly larger in the female dancers (*p* ≤ 0.05). The shoulder and pelvic rotation showed an almost contrary picture.

## Discussion

The aim of the present study was to show the upper body statics in selected dance-specific and dance-non-specific postures, taking gender into account. All dancers stand more upright than non-dancers. The result of this comparison can be explained by the technical requirements of Latin dance [4, 12, 16].

The female dancers of the present study showed smaller torso lengths D and S than the subjects of Ohlendorf et al. [15, 18], while the male dancers showed a larger torso length D and a smaller torso length S when compared to those of Ohlendorf et al. [14, 18]; this can be attributed to the design of the dance figures and the overall aesthetic impression [2]. The significant difference in the lumbar bending angle in all postures was to be expected as females, generally, have a stronger lordosis than men, however, compared to Ohlendorf et al.'s studies [14, 15, 18, 25], this difference is not clinically relevant. Compared to the normal values from Ohlendorf's studies' [14, 15, 18], it can be seen that the male dancers have a lower thoracic bending angle, as well as a lower lumbar bending angle, than the non-dancing subjects [14]. The flattening of the lumbar bending angle in posture 2 compared to posture 1 is considered physiological [23]. It is interesting to note that the value of the lumbar bending angle in the female dancers in posture 4 (wearing dancing shoes with a 7 cm heel barely changes and even remains below the initial value of posture 1. Such a high heel creates a different traction on the ventral and dorsal side of the pelvis. Whether the female dancers in this study are sufficiently flexible or muscularly strong enough, in contrast to what Baumann [4] describes, to compensate for the heel height of the shoe in the lumbar spine, cannot be conclusively clarified here [24]. The male dancer´s heel of 3.6 cm (on average) also has no effect on the lumbar bending angle. Both sexes show a flatter lumbar bending angle in posture 5 than in posture 1; this can be explained by the requested straightening of the spine in dance [2].

When looking at posture 5, a clearly greater spinal rotation of the male dancers (vertical axis) and a decrease in the kyphosis angle can be seen compared to the female dancers; this can most likely be explained by the technical execution (choice of standing leg) and the heel height of the shoes. With high heels, the body automatically compensates for the resulting pelvic tilt and the resulting increased lordosis angle by increasing the kyphosis angle [25].

Considering the shoulder parameters, a significant gender-specific difference was measurable in the area of the shoulder blade distance. This result coincided with the results of Ohlendorf et al. [14, 15, 18]. Due to the requested harmonious couple effect, when partners dance together in which the female is usually smaller and narrower than the male it was found that only the distribution of the shoulder stance values in the males showed a strong similarity to the values of Ohlendorf et al. [14, 18]. In the habitual posture, the subjects of this study, similar to the subjects of Ohlendorf et al. [14, 18], stood with the right shoulder being more dorsal than the left. When looking at the parameters evaluated as a whole, it is noticeable that the males in this study rotate further in the scapula area than the male subjects of Ohlendorf et al.’s studies [14, 15, 18]. The significance of the area of the left shoulder stance angle could result from the greater need for stability in the shoulder blade and arm which the males requires to lead their partners [26].

The non-significant difference in the pelvic distance opens up different courses of discussion. On the one hand, the pelvic distance was not considered in relation to the BMI or abdominal girth in this study, so it cannot be conclusively clarified whether males showed a relative, wider distance between the two SIPS than females. From a purely anatomical point of view, females have a wider pelvis than males [23]. The comparison with Ohlendorf's norm values shows that the female dancers in the present study have a pelvis that is almost more than one centimetre narrower than that of non-dancers [14, 18]. This could provide evidence why Latin American dance is dominated by small females. Male dancers also showed a narrower pelvis than non-dancing males, but this is only a small difference (5 mm) compared to Ohlendorf et al.’s study [14]. Just as with Ohlendorf's norm values, the dancers in this study the left side of the he pelvis stood higher [15, 18]. This may have been due to anatomical pathological patterns, such as a leg length difference or scoliosis, however, none of the dancers reported such a condition. All the dancers were found to rotate minimally around the transverse axis with the right side and, therefore, where found to stand more ventrally in pelvic torsion with the right side of the pelvis, which is almost similar to Ohlendorf et al.’s normal values [14, 15, 18].

Overall, a slight deviation of the frontal trunk decline to the left side could be observed in P1; Ohlendorf et al. [14, 18] found only a slight deviation to the right between 21 and 30 in males, with a slight deviation to the left was found in all females when establishing the normal values. Whether the deviation of this study shows a dancer-specific picture or only reflects the gender distribution of the subject collective cannot be conclusively clarified at this point. Moreover, the deviation studied is not clinically relevant.

When analysing the differences between the right shoulder blade being positioned higher in P2, and the shoulder was found to be positioned more caudally, evidenced by the shoulder stance angles in P5, it should be noted that these are different measurement parameters. The shoulder blade stance is used to analyse the position of the shoulder blades in comparison to the horizontal, whereas the shoulder stance angle analyses the position of the entire shoulder and, specifically, the angle between the neck slope and the horizontal. They are also different postures, especially with respect to the area oft he leading arm. It is quite conceivable that the test persons automatically caudalised the right shoulder, in particular, when assuming the dance posture. Tightening the centring, adducting shoulder muscles helps to achieve a correct dancing posture as well as an aesthetic presentation of the neck and head area.

## Limitations and outlook

This study is the first to be conducted in this regard in dance sport. Posture is very important in dance. The chosen method is a reliable way to measure athletes specifically and on site. However, there are some limitations to be mentioned: During the measurement, due to practicability, no uniform measurement time was observed. It is possible that physical exhaustion may influence the upper body statics to a certain extent. The reproducibility of the measurement may also be limited by the thickness of the skin and the resulting displacement of the markers [19]. The measurement system showed almost no usable data for the shoulder stance angles measured in posture 3; this was due to the calibration of the scanner which calculates this angle based on the hanging upper extremities and scapulae in the normal position [14, 15, 18, 19]. In posture 3, the arms were held in a circle above the head and thus the scapulae were in elevation which resulted in errors, because the markers are glued down to the skin and the scapula as skeletal structure moves underneath the skin during movements. Therefore, the results of the scapula kinematics data in P3 should not be used or at least be interpreted with great caution. Further, one can only measure how much the skin and musculature deform over the scapula if the arm moves upwards, but not scapular angles or motion. In addition to that, it cannot be completely ruled out that the results have been influenced in P2 and P5 due to this problem, too. There have been many attempts to find a solution but without success so far.

## Conclusion

Performing LD changes the static parameters of the upper body posture. This study is an approach to better understand the involved muscular structures in LD. Further projects are required to analyse the field of dance even more thoroughly in terms of sports medicine. With regard to future research, it is important to stimulate practical considerations on compensatory training. Furthermore, it would be interesting to research to the extent to which physical exhaustion affects the upper body statics of dancers.

## Data Availability

The datasets used and analysed during this study are available from the corresponding author on reasonable request.
